# Employing Nanostructured Scaffolds to Investigate the Mechanical Properties of Adult Mammalian Retinae Under Tension

**DOI:** 10.3390/ijms21113889

**Published:** 2020-05-29

**Authors:** Kantida Juncheed, Bernd Kohlstrunk, Sabrina Friebe, Valentina Dallacasagrande, Patric Maurer, Andreas Reichenbach, Stefan G. Mayr, Mareike Zink

**Affiliations:** 1 Soft Matter Physics Division and Biotechnology & Biomedical Group, Peter-Debye-Institute for Soft Matter Physics, Leipzig University, Linnéstr. 5, 04103 Leipzig, Germany; kantida.juncheed@medizin.uni-leipzig.de (K.J.); bkohl@uni-leipzig.de (B.K.); valentina.dallacasagrande@gmail.com (V.D.); 2 Paul Flechsig Institute for Brain Research, Leipzig University, Liebigstr. 19, 04103 Leipzig, Germany; reia@medizin.uni-leipzig.de; 3 Division of Surface Physics, Department of Physics and Earth Sciences, Leipzig University and Leibniz Institute of Surface Engineering (IOM), Permoser Str. 15, 04318 Leipzig, Germany; sabrina.friebe@iom-leipzig.de (S.F.); stefan.mayr@iom-leipzig.de (S.G.M.); 4 Institute of Food Hygiene, Faculty of Veterinary Medicine, Leipzig University, Augustusplatz 10, 04109 Leipzig, Germany; patric.maurer@mri.bund.de

**Keywords:** retina, nanostructured scaffolds, tissue mechanics, nanotubes, tissue stretcher, tissue elasticity, porcine eyes

## Abstract

Numerous eye diseases are linked to biomechanical dysfunction of the retina. However, the underlying forces are almost impossible to quantify experimentally. Here, we show how biomechanical properties of adult neuronal tissues such as porcine retinae can be investigated under tension in a home-built tissue stretcher composed of nanostructured TiO_2_ scaffolds coupled to a self-designed force sensor. The employed TiO_2_ nanotube scaffolds allow for organotypic long-term preservation of adult tissues ex vivo and support strong tissue adhesion without the application of glues, a prerequisite for tissue investigations under tension. In combination with finite element calculations we found that the deformation behavior is highly dependent on the displacement rate which results in Young’s moduli of (760–1270) Pa. Image analysis revealed that the elastic regime is characterized by a reversible shear deformation of retinal layers. For larger deformations, tissue destruction and sliding of retinal layers occurred with an equilibration between slip and stick at the interface of ruptured layers, resulting in a constant force during stretching. Since our study demonstrates how porcine eyes collected from slaughterhouses can be employed for ex vivo experiments, our study also offers new perspectives to investigate tissue biomechanics without excessive animal experiments.

## 1. Introduction

Mechanical properties of cells and tissues play a crucial role in many biological processes, ranging from embryologic development to cancer progression [1,2,3,4,5,6]. Hardy understood, but of major importance for human vision and treatment of eye diseases is the viscoelasticity of the mammalian retina [7,8,9,10]. The retina is a specialized forebrain part taken out to the periphery [11]. It comprises a layered structure of neurons and glial cells with the aim to detect the light, transforming it into neural impulsation and transferring the latter to the brain [12]. Glial cells, the so-called Müller cells, span the retina from the retinal inner surface where the vitreous body is located to the opposite side where rods and cones are found [13,14,15]. Here, the retina attaches to the retinal pigment epithelium. Beside its physiological function, physical properties of the retina and its components play an important role. As shown by Franze et al., Müller cells act as optical fibers guiding the light through the retina to the neurons on the backside [16]. Additionally, the mechanical properties strongly influence pathology and diseases such as diabetic macular edema, macular holes, retinal tears and retinoschisis, which are the major cause of blindness [17,18,19,20,21,22]. A symptom of all these diseases is a change in biomechanical properties of the retina or the retinal pigment epithelium (RPE). Here, rupture of the retina occurs, holes can form, or the entire retina detaches from the underlying RPE, e.g., during wet macular degeneration from which millions of people are suffering all over the world. Here, formation of new blood vessels within the RPE cause tissue swelling, resulting in shear forces, and subsequent tissue rupture. Furthermore, proliferative retinal pathology such as proliferative diabetic retinopathy (PDR) or proliferative vitreoretinopathy (PVR) [23], is characterized by formation of cellular membranes which can cause tensile forces acting on the retina (“traction amotio”). During progression, retinal detachment can occur, which is difficult to prevent in later stages. Consequently, blood supply is hindered, and retinal tissue degradation occurs. 

Besides medical treatment, surgical intervention of the retina is the therapy of choice. However, also here retinal mechanical properties can strongly intervene positive outcome: The retina is located in the spherical eyecup and always under mechanical tension. Thus, it tends to detach and enrolls once the surgeon cuts into the tissue. Difficulties with reattachment and local tissue degradation can be a severe negative side effect [24,25,26]. Additionally, the ways in which forces are applied by the surgeon to e.g., peel the retina on its inner surface to remove pathologically formed membranes during PDR and PVR are determined by the experience of the surgeon itself. Thus, new strategies are necessary to understand the viscoelastic properties of the retina and their impact on disease and surgery.

To quantify the local mechanical properties of retinae ex vivo, scanning force microscopy has been employed to map tissue elasticity on the retinal surface [27], while acoustic radiation force optical coherence elastography offers the possibility to study the mechanical behavior of the different retinal layers with high spatial resolution and sensitivity as reported by Qu et al. [28,29]. However, in all studies tissue preservation, structural integrity, as well as drying effects taking place ex vivo are still an unsolved tasked. To overcome these difficulties, Shahbazi et al. proposed a noninvasive method to estimate the elastic modulus of the retina-choroid complex for age-related macular degeneration in the human eye using sequential ultrasound imaging [30]. In contrast to optical coherence elastography, here, the spatial resolution is reduced, and statistical significance of the results obtained from different patients was reported to be low.

The mammalian retina of pigs is similarly composed as in humans [31,32]. It is vascularized and exhibits an entire thickness of around 220 µm [31,33]. Thus, the porcine retina represents a good testbed to study retina mechanics and relate the results to humans. To overcome the above-mentioned difficulties of adult tissue preservation ex vivo mentioned above, we have shown previously that TiO_2_ nanotube scaffolds are ideal scaffolds for long-term organotypic culture of adult neuronal tissues including the mammalian retinae for at least 14 days [34,35]. Here, the tissue strongly binds to the nanotube surface and the application of adhesive agents is not required. Based on these nanotube scaffolds, we developed a tissue stretcher in which the retina is cultured on two adjacent nanotube scaffolds to investigate the deformation behavior in terms of force-distance relations during tension of the retina ex vivo. By pulling one scaffold apart, a self-designed force sensor determines the forces as function of displacement for different stretching velocities. 

Many diseases are characterized by retinal detachment in which the retina on its photoreceptor side disrupts from the underlying retinal pigment epithelium. To mimic the situation of force exertion on the retina’s photoreceptor layer, we attached the retina on the photoreceptor side to the nanotube scaffolds and applied a loading force on it which results in a displacement propagating from the photoreceptor side through the retina to the opposite surface where the ganglion cell layer is located. In combination with finite element calculations, we obtained a displacement rate dependent mechanical response dominated by the time scales of viscoelasticity as well as the relaxation of internal structural degrees of freedom. 

To this end, our setup and the very sensitive force sensor in combination with fluorescence microscopy images of retina structures can shed more light on the ability of adult retinal tissue to withstand external forces, while providing quantitative data pertaining to forces the retina can withstand without irreversible damage, and if damage occurs, which retinal layer seems to be the “weak link” during rupture.

## 2. Results

### 2.1. Calibration of a Self-Designed Tissue Stretcher

**Figure 1:**
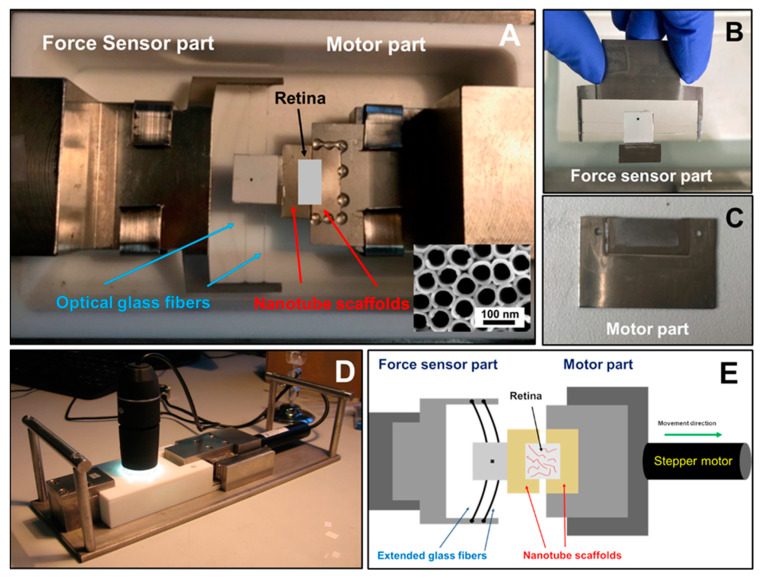
(**A**) Setup of our self-designed tissue stretcher: force sensor and motor parts of the tissue stretcher. The entire device is mounted within a Teflon mold in which culture medium is filled. The position of the retina is marked by the white rectangle. (**B**) For the force sensor part, a printed rectangular dot on the white sticker was glued on top of the stainless-steel plate in order to track the position during stretching and thus the elongation of the glass fibers. (**C**) The motor part composed of a nanotube scaffold attached to a stainless-steel holder is connected to stepper motor which moved apart during stretching. (**A‒C**) Force sensor and motor parts were attached to two adjacent nanotube scaffolds to support adhesion and organotypic culture of the retina explant on top. (**D**) Outside view: A camera was attached to monitor position alteration of rectangular dot on the stainless-steel plate while the stepper motor moved. (**E**) Sketch of the working principle: During stretching, the stepper motor moved one nanotube scaffold apart with the retina on top. When the retina expends, the glass fibers of the force sensor extend, resulting in a position alteration of the rectangular dot in the center of the force sensor which was imaged with a camera positioned above the tissue stretcher.

We employed a self-designed tissue stretcher to investigate the mechanical properties of adult porcine retinae extracted from pigs from a slaughterhouse under tension. To this end, we placed a freshly prepared retina on two solid TiO_2_ nanotube scaffolds which were initially located next to each other without a gap in between with the retina positioned at the interface of the two scaffolds (Figure 1). During tissue stretching, one nanotube scaffold is stationary and the other one is pulled away by a stepping motor with constant velocity. The stationary part is connected to a self-designed force sensor. The force sensor is composed of a nanotube scaffold attached to glass fibers of 200 µm diameter which act as springs and elongate when the retina on the nanotube scaffolds is stretched.

After determining the spring constant of the glass fibers prior to retina stretching (see Materials and Methods), forces acting during deformation can be derived from Hooke’s law. 

### 2.2. Force-Distance Behavior of the Retina during Stretching

**Figure 2:**
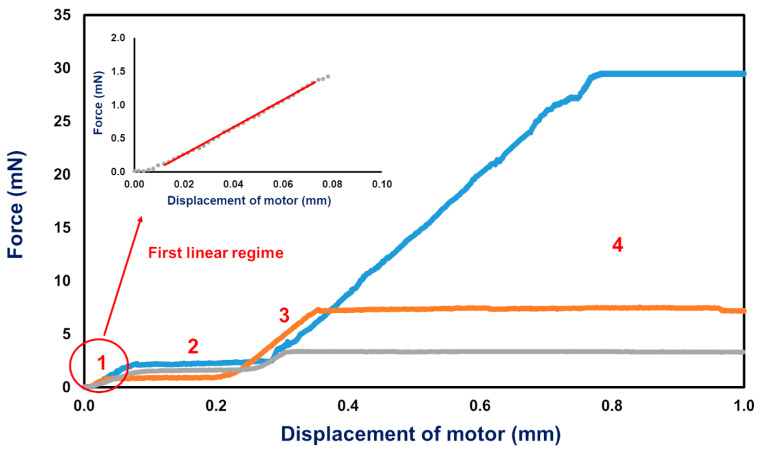
Force-Displacement curves of three individual retina stretching experiments with different stretching velocities: 0.1 µm/s (blue), 0.5 µm/s (orange), and 2.0 µm/s (grey). The inset shows the magnification of the FD curve for small displacements for a displacement rate of 2.0 µm/s. The FD curves display four different regimes: (**1**) A linear force increase for small displacements; (**2**) yielding and plastic deformation at constant force; (**3**) a second force increase for larger displacements followed by (**4**) another constant force plateau.

After positioning the fresh pig retina on the tissue stretcher at the interface of the two adjacent nanotube scaffolds (Figure 1E), the motor pulls one of the scaffolds away with a constant velocity, resulting in a force acting on the retina and stretching of the tissue. Representative examples of the resulting force-distance (FD) curves for three different stretching velocities (0.1 µm/s, 0.5 µm/s and 2.0 µm/s) are shown in Figure 2. 

**Figure 3:**
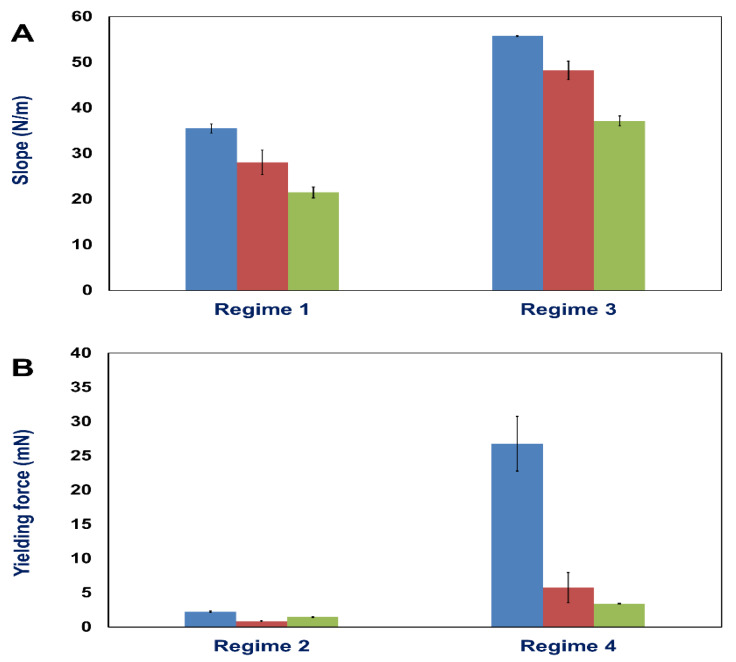
(**A**) Slopes of force-distance curves within regime 1 (elastic constants) and regime 3; and (**B**) yielding forces (maximum forces) at the beginning of regime 2 and 4 for different displacement rates: 0.1 µm/s (blue), 0.5 µm/s (orange), and 2.0 µm/s (green). The numbers give mean values and standard deviations (SD).

The FD curves displace four different regimes: (1) For small displacements below 0.05 mm, the force increases linearly, characteristic for elastic deformation. From the slope the elastic constant (in terms of the proportionality constant between the measured forces and distance) of the retina can be determined. This linear regime is preceded by a non-linear force increase for displacements below 0.01 mm (see inset in Figure 2) which we attribute primarily to lose clamping of the retina at the interface of the two nanotube scaffolds but also entropic contributions of the biopolymer networks. (2) The linear force increase is followed by a yielding point after which a saturation and a constant-force regime become present (regime 2). (3) Subsequently, in regime 3 the force increases linearly again with a higher slope compared to the first linear force increase of regime 1. Also, here, a transition to a second saturation regime (regime 4) with a constant force becomes visible for large deformations. Such FD behavior is seen for all employed stretching velocities, while the slopes of regimes 1 and 3, as well as the yielding forces change with displacement rate (Figure 2 and Figure 3). A comparison of the slopes of regimes 1 and 3, as well as of yielding forces for different displacement rates, are shown in Figure 3.

In fact, for a displacement rate of 2.0 µm/s, an elastic constant of (21.5 ± 1.2) N/m was found for the first linear regime, while for a slower velocity of 0.1 µm/s, the elastic constant was higher with a value of (33.5 ± 4.7) N/m. Also, the yielding forces increased from (1.5 ± 0.1) mN for 2.0 µm/s to (2.2 ± 0.1) mN for a velocity of 0.1 µm/s. To this end, the maximum forces the retina can withstand without irreversible damage are velocity dependent, as well as the corresponding displacements at which yielding occurred (Figure 2). Similar results become visible for regime 3 (Figure 2 and Figure 3) which showed the largest force increase for small displacement rates, as well as the smallest yielding point/constant force of regime 4. Thus, the elastic constants as well as yielding points, show a displacement rate dependence, which is beyond simple rheological models for viscoelasticity. However, we will come back to this aspect and the physical meaning in the discussion section. Within this context we would like to point out that our experiments show very reproducible results with small errors of the obtained slopes and yielding forces. Moreover, the retina was strongly attached to the nanotube scaffold (see Materials and Methods) and slippage of the tissue during the experiment was never observed.

### 2.3. Determination of Shear Modulus Assisted by Finite Element Calculations

In case of a pure elastic tensile extension of the retina during stretching, a conversion of the force into stress and the displacement into a dimensionless strain would result in a linear stress-strain dependency from which the slope describes the Young’s modulus of the retina. However, the initial sample length (viz. the area of the tissue where stretching takes place) is immeasurably small because the two nanotube scaffolds with the retina on top are initially touching each other. Thus, it is not possible to normalize the displacement to calculate the strain. Moreover, since the retina is stretched by pulling onto the bottom of the retina perpendicular to the cross-sectional area along which the displacement is expected, the deformation is not pure uniaxial strain within the substrate plane, but contains additional shear components. Thus, we performed finite element simulations to gain more details on the displacement field and the material properties of the retina. 

**Figure 4:**
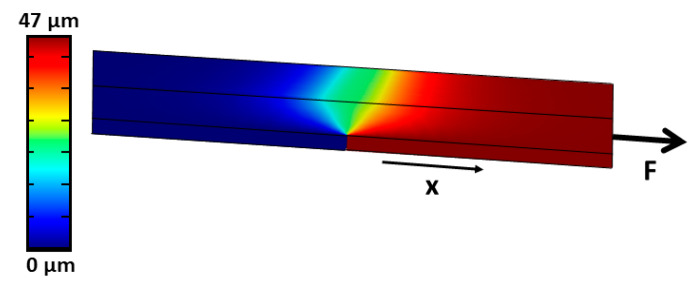
Finite element simulation of a viscoelastic material (retina) on top of two adjacent titanium plates. While the left plate is fixed with position constrains, a loading force of 1.0 mN in x-direction is acting onto the right sidewall of the right titanium plate, resulting in a displacement in x-direction of 47 µm for a chosen retinal Young’s modulus of 760 Pa. The displacement in x-direction is color-coded from blue (no displacement) to red (maximum displacement). It can be seen that the retina material is only stretched – viz. a shear deformation is visible – close to the gap where the two scaffold materials are pulled apart. At all other positions the retina is strongly attached to the scaffolds and moves according to the underlying substrate.

Within the simulation, the same experimental conditions were employed as obtained in the experiments. To gain the effective Young’s modulus of the retina from our experiments, we varied the Young’s modulus within the simulation iteratively until we got the same displacement for a certain loading force found experimentally within the first linear (viz. elastic) regime in the FD curves. As an example, Figure 4 shows the displacement field when a load of 1.0 mN acts onto the substrate to stretch the retina on top. Here the total displacement in x-direction is 47 µm, when a retina’s effective Young’s modulus of 760 Pa is used. As can be seen from the displacement field in Figure 4, a shear deformation within the retina becomes visible at the position above the gap of the two nanotube scaffolds.

In summary, we found an effective Young’s modulus for the first linear increase (regime 1) of around 1270 Pa for stretching velocities of 0.1 µm/s, approximately 1000 Pa for 0.5 µm/s and 760 Pa for 2.0 µm/s, respectively. Thus, the retina behaves softer for faster loading rates. Moreover, even for small displacement rate variations of less than one order of magnitude, a remarkable change in material properties become visible. 

### 2.4. Fluorescence Imaging of the Retina during Deformation

**Figure 5:**
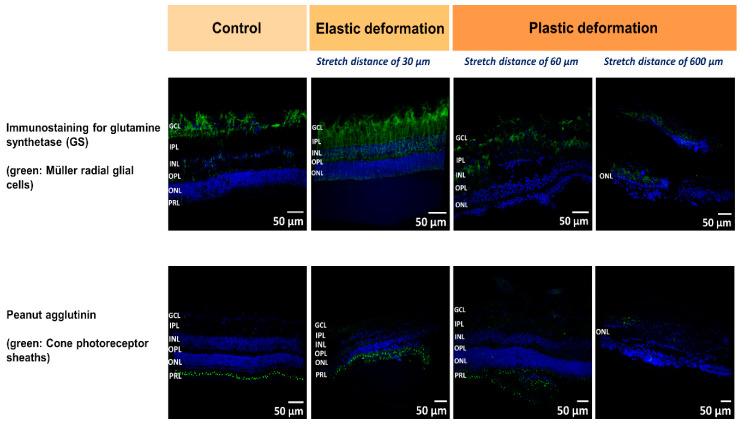
Labeling of porcine retinae to visualize cell processes (immunohistochemistry) and nuclei (blue staining by Hoechst 33342) indicating the retinal layers; GCL, ganglion cell layer; IPL, inner plexiform layer; INL, inner nuclear layer; OPL, outer plexiform layer; ONL, outer nuclear layer; and PRL, photoreceptor layer. Retinal tissue was labeled after a 0.1 µm/s deformation over a stretch distance of 30 µm (elastic deformation) and 60 µm or 600 µm (plastic deformation), respectively; left column: controls. Müller (radial glial cell) processes were labeled by immunostaining for glutamine synthetase (green, top row). The sheaths of cone photoreceptor cells were labeled by peanut agglutinin (green, bottom row).

In order to get a first impression how the retina deforms during stretching, we performed immunostaining to visualize retinal layers during a 0.1 µm/s deformation after a stretch distance of 30 µm (elastic regime: reversible deformation), 60 µm (yielding: the retina begins to rupture), and 600 µm, respectively. Here, the retina was fixed and removed from the stretcher device. Subsequently, we employed different fluorescently labelled antibodies to image Müller radial glial cells and cone photoreceptor cells compared to the un-stretched retina (control) (Figure 5). The control images clearly show the five characteristic subretinal layers: (i) outer nuclear layer (ONL); (ii) outer plexiform layer (OPL); (iii) inner nuclear layer (INL); (iv) inner plexiform layer (IPL); (v) ganglion cell layer (GCL) (for more details on retina morphology see ref. [11,33,36]).

## 3. Discussion

Even though it has been known for decades that changes in the mechanical properties of the retina are often related to eye diseases which can result in loss of vision [37,38,39,40,41], up to now, the possibility for a detailed characterization of eye mechanical properties has been restricted. Rodents are usually the animals of choice for in vitro investigations. However, the avascularized retinae of Guinea pigs and rabbits display the properties of the human eye only to a limited extent [42,43], while the vascularized retinae of rat and mice are thinner compared to humans and exhibit large thickness distributions suggesting that the arrangement of blood vessels might be different from human retinae [42,44,45]. Alternatively, the size and structure of the vascularized porcine retina are very similar to the human retina [31,32,46,47], thus porcine eyes offer a good testbed to study the mechanical properties ex vivo. In fact, Wollensak et al. employed a biomaterial test machine to stretch retinal strips of porcine eyes horizontally [48]. From the resulting stress–strain relationships, an average Young’s modulus of 10^5^ Pa was reported, while for an increased strain rate, the Young’s modulus increased as well. However, the retinal strips dried during stretching which was expected to stiffen the material compared to fresh tissue. Furthermore, Chen and coworkers used uniaxial tension tests for porcine retinae and determined an elastic modulus around 5 × 10^4^ Pa [47]. They also found that the direction in which the sample was cut significantly affected the tensile stiffness. In later studies, they explained that the retina elasticity depends on the size and orientation of the blood vessels inside the retina [49]. Worthington et al. obtained a Young’s modulus of porcine retina under compression of 10.5 kPa [50], similar to tension experiments mentioned above. Employing avascularized guinea pig eyes, Franze et al. used scanning force microscopy (SFM) to determine the local mechanical properties of the retinae, in contrast to the global mechanical properties investigated in the above-mentioned studies. Here, an elastic modulus ranging from 940 Pa to 1800 Pa was found, depending on the indentation position of the SFM tip [27]. For other avascularized retinae of rodent animals, several publications show Young’s moduli in the range of 2 kPa to 16 kPa, depending on the experimental conditions [27,28,50].

Studies on retinal layer elasticity by Qu et al. showed that the ganglion cell layer (GCL) of porcine eyes is softest with a Young’s modulus of (1.33 ± 0.37) kPa – similar to the Young’s moduli observed in our experiment for regime 1 – while the photoreceptor layer (PRL) and ONL are hardest compared to other retinal layers with a Young’s modulus of (25.9 ± 7.36) kPa [29]. In addition, Weber et al. reported a similar trend. Here, the photoreceptor layer/outer nuclear layer (PRL/ONL) and inner nuclear layer (INL) provided the highest stiffness with elastic moduli of 330 Pa and 216 Pa, respectively, while the outer plexiform layer (OPL), inner plexiform layer (IPL), and the GCL were softer with an elastic modulus of 199 Pa, 153 Pa, and 157 Pa, respectively [51]. Both nuclear layers (ONL and INL) were significantly stiffer than the plexiform layers (OPL and IPL) because the density of cell nuclei is considerably higher in the nuclear layers than in the plexiform layers. Differences in cell body densities undoubtedly contributed to local stiffness in retinal tissues [51]. 

In our present study, we developed a tissue stretcher with a self-designed force sensor to quantify the forces acting during uniaxial retina deformation under tension. Additionally, we show how fresh adult porcine eyes collected from slaughterhouses can be employed to investigate eye mechanics ex vivo. After validating the working principle of our stretcher device, we studied the force–distance behavior and displacement rate dependence of porcine retinae deformation. Even though biological tissues are never the same for different animals since they exhibit small variations in size, cellular and molecular structure, we found that the results in terms of the measured force–distance (FD) curves are remarkably reproducible with small standard deviations of elastic constants and yielding forces. In every stretching experiment, we observed four different deformation regimes (Figure 2): (1) a linear force increase characteristic for elastic deformation [4,5,52,53], (2) a transition to a maximum force (yielding force) followed by a constant force which is supported to characterize tissue failure in terms of rupture and “fluid-like” behavior as not only found in tissues, but also glassy materials [54,55], (3) a second force increase, and (4) a saturation regime of constant force for large displacements. 

By using finite element calculations for a more detailed data analysis, we determined the Young’s modulus of the retina during elastic deformation (regime 1). We found values in the order of (760–1270) Pa depending on the deformation velocity. Interestingly, for an increasing displacement rate from 0.5 µm/s to 2.0 µm/s, we observed a decrease of the determined Young’s modulus, as well as a decrease in yielding force succeeding the elastic deformation. Also, for the second linear force increase (regime 3), the slope decreases for higher stretching velocities, as well as the maximum force plateau following the force increase. The observation that yielding occurs faster, viz. for smaller displacements or strains when a higher deformation rate is applied, is usually found in many biological and classical (hard) materials [10,48,49,52,56,57]. However, usually soft as well as hard matter behave stiffer for high strain rates due to a decreasing relevance of flow and rupture happens faster, i.e., for smaller strains. Besides, even though the employed displacement rates vary only of the order of one magnitude, a strong displacement rate dependence of both elastic constants and yielding forces becomes present.

Considering tissue as a liquid-like material which can also exhibit glass-like behavior, we surmise that the observed high displacement rate dependence can be attributed to non-Newtonian flow during viscoelastic deformation within regime 1. Here, athermal effects contribute, viz. the employed displacement rates $\dot{\gamma }$ are too high that internal structural adjustments towards local equilibrium, viz. sampling around the new local minimum free enthalpy configuration does not take place sufficiently on the experimental timescale given by $\frac{1}{\dot{\gamma }}$. This scenario has been demonstrated by us for straining glassy model systems in molecular dynamics computer simulations [54]. Additionally, as described by Chen et al., in biological systems in which chemical bonds such as hydrogen bonds play a role, friction forces decrease with increasing deformation velocities due to the reduced time available for formation [58]. Even though in the retina numerous covalent bonds are expected to dominate structural integrity besides hydrogen bonds, it is not the bond type which determines friction, but the time scale on which bond breakage and reformation take place during deformation. Thus, for fast displacement rates, the potential for bond reformation is expected to become insufficient on the given time scales and dissipation in terms of internal friction becomes a less dominating factor for the material’s stiffness. Mind that this is accord with our interpretation of the displacement rate dependence by athermal/non-ergodic contributions. In future experiments, we propose to significantly lower the displacement rate and identify the timescale at which local ergodicity is reestablished. This will provide us with a measure of the internal structural relaxation time scale of our retinae.

At the microscale, we expect that local heterogeneities and the layered structure of the retina determine the observed FD behavior. Thus, we employed immunostaining to visualize retinal layers before and during stretching. During elastic deformation after a displacement of 30 µm, we observed that the retinal structure was preserved compared to control, while the entire thickness of the retina was reduced during tissue expansion (Figure 5, second row). Less Müller cell nuclei from the GCL become visible and the thinning of the tissues seems to occur in line with a delocalization of nuclei from the ONL and INL which might relocate towards the center of the retina, viz. the IPL. As observed by Lindqvist et al., mechanical stimulation and stress cause reactive changes of Müller cells which became visible by the increased Müller glia staining (immunostaining for glutamine synthetase) in Figure 5, (upper row after 30 µm stretch) [59]. We would like to point out that the retina was fixed on the nanotube scaffolds after stretching. For staining and imaging, however, the tissue had to be removed. Thus, the entire stretching behavior and displacement of elastically deformed tissue might not be visible due to relaxation of structural component to their initial position.

In the linear force regime, we observed that Müller cells start to tilt during shear deformation, as well as delocalization of the nuclei occurred, which most likely result in friction forces between these tissue components and are expected to contribute to the viscoelasticity and the influence of friction mentioned above. 

When the transition from the linear force increase to the constant force regime took place (stretch distance after 60 µm, Figure 5), our images of the retina revealed that, first, the ONL and INL maintained their structure while the thickness of IPL/GCL were reducing compared to control. Second and most striking is rupture and crack formation found in the IPL/GCL layer (Figure 5, third row). Since the force remained constant for around 200 µm stretching distance, we propose that sliding at the interface of the crack and ruptured tissue layers occurred, during which stick and slip events reached equilibrium [58]. Even though the acting forces within the ONL and INL are expected to be larger compared to the IPL/GCL since the nuclear layers are closer to the nanotube sheets which move apart (viz. the displacement is larger in the nuclear layers compared to the layers closer to the upper retinal surface – compare displacement field in Figure 4), the nuclear layers are mechanically more stable and can withstand larger strains in line with Qu et al.’s and Weber et al.’s findings mentioned above. 

In a further experiment in which we stopped deformation after 600 µm (end of the second linear force increase), we still see an intact ONL and no rupture, while the other layers of the retina were not detectable anymore and the structure was fully disrupted (Figure 5, right row). Similar to the constant force regime 2, here, an equilibration between slip and stick events at the interface of ruptured tissue layers is expected resulting in a constant force even for large stretch distances. Taken together, during the first constant force regime the interconnections between the retinal components were still present even though slippage of ruptured layers occurred. In the preceding second force increase (regime 3), strain hardening of the ECM network holding together the different layers might take place which finally ends in complete detachment of the nuclear layers from the IPL/GCL and lapse with constant force (regime 4). We would like to mention that the constant force regimes cannot be attributed to slippage and detachment of the retina from the nanotube scaffolds since here the force is expected to stay constant and finally drop for large stretch distances. In contrast, after constant force regime 2 we saw a second force increase (regime 4) which would not be present if the retina detached from the scaffolds. Besides, after stretch we could only detach the retina from the nanotube scaffolds by strongly pulling on it with tweezers and peeling if off.

To get more insight into the contribution of structural changes during deformation, future studies will focus on the detailed characterization of retinal layer biomechanics in combination with image analysis techniques. Since biomechanical variations of the retina and the underlying retinal pigment epithelium are the origin of rupture, hole formation and retinal detachment, it is of clear importance to understand what forces the retina can withstand without irreversible damage to propose the progression of eye diseases for patient with early retinal dysfunction. Our setup, in combination with the application of porcine eyes from slaughterhouses, offers a new approach to study the influence of acting forces on eye diseases, while it also allows for investigations of new drugs and reagents on retinal elasticity. 

## 4. Materials and Methods 

### 4.1. Preparation of Retina Tissue

Freshly extracted eyes from adult pigs 6–7 months old with an average weight of 100–150 kg were collected from the slaughterhouse E. Färber GmbH Großschlächterei & Co. KG in Belgern-Schildau, Germany. Eyes were transported to our laboratory in cell culture plastic tubes filled with cooled phosphate buffered saline (PBS) placed on ice bags in an insulated transportation box. After about 1-h transportation time, eyes were removed from the tubes, placed in a plastic dish with ice cold PBS and all irrelevant tissues around the eyeballs were cut off (yellow fat, muscle, and conjunctive tissues). Subsequently, isolation of the retina was performed in a sterile room dedicated to primary culture experiments. First, the eyeball was shortly dipped in 70% ethanol and washed twice with sterilized PBS before transferring to the Petri dish with PBS. An incision was made behind the ora serrata to release the pressure of the posterior cavity allowing to hold the eyeball easily. Equatorial sectioning of an eyeball was started from about 2 mm behind the cornea by using scalpel, eye scissors and forceps. After removal of the lens, the retina remained attached to the vitreous body. By using forceps, the retina was removed carefully from the underlying pigment epithelium. Subsequently, the remaining vitreous body was gently removed as well.

To perform all experiments with reproducible conditions, the tissue was stamped with a self-designed rectangular tissue stamper to a size of 10 × 6 mm^2^. All tissue samples were stamped horizontally at the same position at the center of the retina close to the macula. Subsequently, the retina explants were placed on the tissue stretcher as described below. Supply with nutrients was ensured with retina culture medium composed of AMES medium (A1420, Sigma-Aldrich, Germany) with 20% horse serum (H 1270, Sigma-Aldrich, Germany) and 0.1% gentamicine (1357, Sigma-Aldrich, Germany).

### 4.2. Working Principle of Tissue Stretcher

Our retina tissue stretcher is based on attachment of the retina to two TiO_2_ nanotube scaffolds located next to each other with the tissue placed on top above the interface of the scaffolds (Figure 1A). One of the scaffolds is attached to a high-resolution linear actuator with stepper motor (Catalogue Number M-228.10S, Physik Instrumente (PI), Karlsruhe, Germany) which moves with constant velocity away from the other scaffold which is fixated (Figure 1C). The gap between the scaffolds is subsequently increased while the retina on top is stretched accordingly (Figure 1E). The stationary scaffold is connected to a self-designed force sensor to measure the forces acting during retina deformation (Figure 1B). A self-written LabVIEW program (National instrument, LabVIEW 2011) controls the motor movement and displacement rate, while a digital USB microscope camera (800× magnification, highest picture resolution 1600 × 1200, Guangzhou Sunshine Electronic Technology, China) images the deformation from the top. The stretcher part including the retina and the force sensor is enclosed in a Teflon mold and filled with culture medium. During the experiment the mold is closed with a cover of the same material; the stretching procedure is then imaged through a camera viewport (Figure 1D). The entire setup is placed in a cell culture incubator at 37 °C and 5% CO_2_. A prerequisite for retina stretching is the attachment of the tissue to a solid scaffold material. Here we used TiO_2_ nanotube sheets as shown in Figure 1A. As we have reported previously, TiO_2_ nanotube scaffolds are ideally suited for long-term culture of adult neuronal tissues such as the retina, as well as brain explants [34,35]. Here, we employed nanotube scaffolds with a nanotube diameter of around 70 nm and an entire thickness of 100 µm. These nanotube scaffolds, which were produced by electrochemical anodization as reported in Dallacasagrande et al. [35], support strong intrinsic adhesion of the tissue to the surface without the need of any glues or adhesive proteins.

### 4.3. Working Principle of the Force Sensor

A self-designed force sensor was employed to determine the forces acting during retina stretching. As described above, prior to stretching, the retina was placed on two adjacent nanotube scaffolds. While one scaffold was attached to a stepping motor, the stationary part belongs to the sensor. In a first fabrication step, a stainless-steel plate of size (10 × 10) mm^2^ was glued on two parallel aligned glass fibers of diameter 200 µm (Thorlabs, Germany). A rectangular dot on white background sticker was glued on top of the stainless-steel plate to track the alteration of glass fibers position during the experiment (black dot in Figure 1B). Prior to retina stretching, a nanotube scaffold sheet was glued on top of the stainless-steel plate and positioned directly next to the nanotube scaffold of the motor part. When the nanotube scaffolds were separated by the motor movement and the retina was stretched, the glass fibers acted as springs and elongated. During the stretching process, the elongation was imaged with a digital USB microscope camera mounted above the stretching device which tracked the position change of the rectangular dot on top of the stainless-steel plate. A self-written LabView routine used the alteration of the rectangular dot position to determine the extension of the glass. Based on the spring constants of the glass fibers, forces can directly be derived from Hooke’s law.

To determine the spring constant, we added extra masses of brass wires with known values (150‒2200 mg) to the force sensor gadget and measured the elongation of the glass fibers with a camera as a function of gravitational force. After calculating the acting gravitational forces from Newton’s law, we obtained a linear force increase as function of glass fiber extension). From the slope, the spring constant can directly by derived and we obtained a value of (54.96 ± 0.52) N/m. For control, the experiment was repeated with the TiO_2_ nanotube scaffold attached to the force sensor as employed in our stretching experiment. Subsequently, extra masses were added to the force sensor to determine the spring constant. The result displayed almost no effect with an increase in the spring constant value of less than 1.5%.

### 4.4. Experimental Procedure of Retina Deformation

After extraction and punching of the fresh retina, the tissue was positioned on the middle of two TiO_2_ nanotube scaffolds with the photoreceptor-side down (Figure 1A). AMES medium with 20% and 0.1% gentamicin was filled within the mold of the tissue stretcher up to a level that it came into contact with the nanotube scaffolds but did not cover the tissue. Because the TiO_2_ nanotube scaffolds are super hydrophilic, fresh medium always diffused to the tissue for sufficient medium supply [35]. After incubating the entire setup inside a cell culture incubator at 37 °C and 5% CO_2_ for 22 h, retinal attachment to the nanotube scaffolds was achieved. We would like to point out that the retinal structure and cell viability are fully maintained on the nanotube scaffolds, while strong adhesion is achieved without the application of adhesive agents [34,35]. In fact, by choosing the “right” nanotube geometry, the retina binds to the nanotube surface within a few hours and even during retina stretching the connection holds and no slippage takes place.

Next, the retina was stretched by tearing one nanotube scaffold away from the other with a stepper motor. The motor is attached to a linear actuator which transforms rotational motion to linear travel; a distance of 1 µm requires a full rotation of 24 equal steps. We employed three different motor velocities (in the following named displacement rates: 0.1, 0.5, and 2.0 µm/s, respectively, and total displacements (viz. the distance of motor movement) up to 1 mm. 

After stretching, the tissue stretcher was removed from the incubator and the retina was fixed on the nanotube scaffolds with 4% paraformaldehyde (158127, Merck, Germany) in PBS for 48 h. Subsequently, the retina was gently removed with forceps. For each experiment new nanotube scaffolds were used. Prior to retina experiments, nanotube scaffolds were cleaned with 70% alcohol while the entire tissue stretcher was deconstructed, autoclaved, and subsequently reconstructed inside the sterile room.

### 4.5. Immunohistochemical Staining of Retina Tissue

Immunohistochemical staining of the porcine retina was applied to visualize retinal structures inside the retina after mechanical testing. The change in cell layer structure inside the retina was revealed by tracking Müller radial glial cells, and cone photoreceptor. After removing the fixed retina from the nanotube scaffolds with forceps, it was embedded in 3% agarose gel in PBS and subsequently segmented by a vibration microtome with slice thickness of 30 µm. Then, 3–5 sliced samples were transferred into 24-well plates and filled with 2 mL of PBS. Next, the samples were incubated with 1 mL of washing buffer (1% DMSO + 0.3% Triton X-100 in PBS) for 10 min at room temperature. After removing the washing buffer, 1 mL of blocking solution (5% of donkey serum in washing buffer) was added and subsequently incubated for 1 h. In the meantime, primary antibodies were diluted to 1:100 in blocking solution. In this study, anti-glutamine synthetase produced in mouse (MAB302, Chemicon, Germany) was used to track Müller radial glial cells, and peanut agglutinin conjugated biotin (L6135, Sigma-Aldrich, Germany) for cone photoreceptor [32,46]. 

After overnight incubation, samples were washed three times with washing buffer for a total of 3 h. For fluorescence detection of all tracking cell types, staining kits with cyanine conjugated dyes (Cy2 and Cy3) with 1:200 in washing buffer were utilized. Cy2 produced in donkey anti mouse (715-225-150, Jackson ImmunoResearch, UK) served to visualize Müller cells incubated with anti-glutamine synthetase. Cy2 conjugated with streptavidin (016-220-084, Jackson ImmunoResearch, UK) was employed for cone photoreceptor visualization. Hoechst 33342 (H3570, Life technologies) was used as a nuclear counterstaining (1:1000 in washing buffer). Negative controls were carried out without primary antibodies in order to ensure specific binding of primary and secondary antibodies. After 2 h incubation in dark room, sections were washed with PBS several times. Finally, slides were mounted on glass coverslip with glycerol and visualized with a laser scanning confocal microscope (fluorescence detection; CLSM 880 NLO Fast Airyscan; Carl Zeiss, Germany).

### 4.6. Statistical Analysis

For a displacement rate (viz. motor velocity) of 0.5 µm/s and 2.0 µm/s, three stretching experiments were performed, while for 0.1 µm/s five stretching experiments were conducted. Raw data obtained from tissue stretcher were used to generate force-distance curves. After calculating the slope of linear regimes and determining yielding forces, we determined the mean values and standard deviation (SD) of these numbers for the three different velocities. 

### 4.7. Finite Element Calculations

Finite element calculations using COMSOL Multiphysics^®^ 5.3 were employed to determine the effective Young’s moduli of the retina during deformation from the experimentally determined forces for various displacements and displacement rates. First, the “model builder” was used to build up two scaffolds onto which the retina is placed. Since both pure titanium and TiO_2_ nanotube scaffolds exhibit Young’s moduli decades larger than the soft retinal tissue [60], we can expect that the scaffolds onto which the retina is placed reveal decades lower deformation than the retina on top. Thus, we did not model the nanotubes but employed pure titanium with material parameters from the Comsol data bank for the material properties of the two modeled scaffolds. The scaffolds comprised the same geometry as employed in the experiments (size of each scaffold: 3 mm width, 10 mm depth, 0.1 mm height). The scaffolds were located next to each other with the retina modeled on top covering the entire surface of the two scaffolds (retina size: 6 mm width, 10 mm depth, 0.22 mm height as found for vascularized pig retinae—see Supplementary Figure S2) [11,31,36]. For the retina, we assumed an almost incompressible viscoelastic material model with a density of 1017 kg/m^3^ [61], and a Poisson ratio of 0.49 [62].

Free tetrahedral structural solid finite elements were defined before generating a fine mesh where the scaffold including the retina was discretized into 93373 elements. Subsequently, we used the “solid mechanics” module with position constrains on one titanium scaffold to keep it fix in all directions and position constrains on the second scaffold in z-direction (see Supplementary Figure S2). Additionally, onto this scaffold a predefined face load with force values found from the linear regimes in the experimental force-distance curves was acting in x-direction to gain the maximum displacement of the scaffold and extension of the retina as function of loading force and effective Young’s modulus. Within the simulation, we iteratively varied the Young’s modulus of the retina for a fixed loading force until we gained the same displacement as found in the experiment.

## 5. Conclusions

Based on TiO_2_ nanotube scaffolds which support adhesion and long-term culture of adult neuronal tissues ex vivo, we showed how these scaffolds integrated into a tissue stretcher with a self-designed force sensor can be employed to study tissue mechanics under tension. In fact, numerous eye diseases are linked to biomechanical dysfunction such as hole formation in the macular or rupture of retinal layers. Here, forces acting on the photoreceptor side of the retina can result in retinal detachment during many eye diseases. To address these issues, we studied the influence of tension acting onto the photoreceptor side which then propagated through the retina towards the opposite surface. We observed a high displacement rate dependence of elastic behavior which can be attributed to the heterogeneous structure of the vascularized retina. For different displacements and displacement rates, we showed that a viscoelastic material such as the retina can behave elastically, or even liquid-like, while fast displacements resulted in decreased stiffening due to reduced friction between structural components. 

Especially in older patients, the vitreous body begins to shrink and pulls away from the retina. When the vitreous body sticks to the retina, macular stretching and irreversible hole formation can follow. For a better understanding of the underlying biomechanical properties, our setup offers the possibility to culture the retina with the ganglion cell layer attached to the nanotube scaffolds to quantify the resulting displacement field and acting forces during tension. Additionally, eye surgeons also inevitably have to apply a force to the retina which can cause severe negative side effects such as rupture. To reduce possible complication during surgery, modern surgery is assisted by computer and robots [63,64,65]. Nevertheless, even highly successful surgery technology is only possible if the forces which the retina can withstand without irreversible damage are known. Thus, a quantification of forces during different load applications is a possible future use of the stretcher device. To this end, the use of nanomaterials that support tissue preservation ex vivo offers various perspectives in future medicine ranging from novel minimally invasive surgery techniques up to drug testing without excessive animal experiments.

## Supplementary Materials

The following are available online at https://www.mdpi.com/1422-0067/21/11/3889/s1, Figure S1: Determination of spring constant, Figure S2: Sketch of finite element calculation of retina stretching.

## Abbreviations

GCL	Ganglion Cell Layer
INL	Inner Nuclear Layer
IPL	Inner Plexiform Layer
ONL	Outer Nuclear Layer
OPL	Outer Plexiform Layer
PDR	Proliferative Diabetic Retinopathy
PRL	Photoreceptor Layer
PVR	Proliferative Vitreoretinopathy
RPE	Retinal Pigment Epithelium
