# Supplementary material for: Employing Nanostructured Scaffolds to Investigate the Mechanical Properties of Adult Mammalian Retinae Under Tension
Source: Int J Mol Sci. 2020 May 29;21(11):3889. doi: 10.3390/ijms21113889 (PMC7313470; doi:10.3390/ijms21113889)
Supplement: Supplementary file 1 [file ijms-21-03889-s001.pdf]

Supplementary Materials

Employing Nanostructured Scaffolds to investigate the Mechanical Properties of Adult Mammalian Retinae under Tension

Kantida Juncheed [1,2], Bernd Kohlstrunk [1], Sabrina Friebe [3], Valentina Dallacasagrande [1,2,4], Patric Maurer [5,6], Andreas Reichenbach [2], Stefan G. Mayr [3], Mareike Zink [1,*]

[1] Soft Matter Physics Division and Biotechnology & Biomedical Group, Peter-Debye-Institute for Soft Matter Physics, Leipzig University, Linnéstr. 5, 04103 Leipzig, Germany; kantida.juncheed@medizin.uni-leipzig.de (KJ); bkohl@uni-leipzig.de (BK); zink@physik.uni-leipzig.de (MZ)

[2] Paul Flechsig Institute for Brain Research, Leipzig University, Liebigstr. 19, 04103 Leipzig, reia@medizin.uni-leipzig.de (AR)

[3] Division of Surface Physics, Department of Physics and Earth Sciences, Leipzig University and Leibniz Institute of Surface Engineering (IOM), Permoser Str. 15, 04318 Leipzig, Germany; sabrina.friebe@iom-leipzig.de (SF); stefan.mayr@iom-leipzig.de (SGM)

[4] Present address: Department of Pediatrics, Weill Cornell Medical College, 1300 York Ave, New York, USA; valentina.dallacasagrande@gmail.com (VD)

[5] Institute of Food Hygiene, Faculty of Veterinary Medicine, Leipzig University, Leipzig, Germany.

[6] Present address: Max Rubner-Institut (MRI), Federal Research Institute of Nutrition and Food, Department of Safety and Quality of Meat, E.-C.-Baumann-Str. 20, 95326 Kulmbach, Germany; patric.maurer@mri.bund.de (PM)

* Correspondence: zink@physik.uni-leipzig.de; Tel.: +49 (341) 9732573; Fax: +49 (341) 9732479

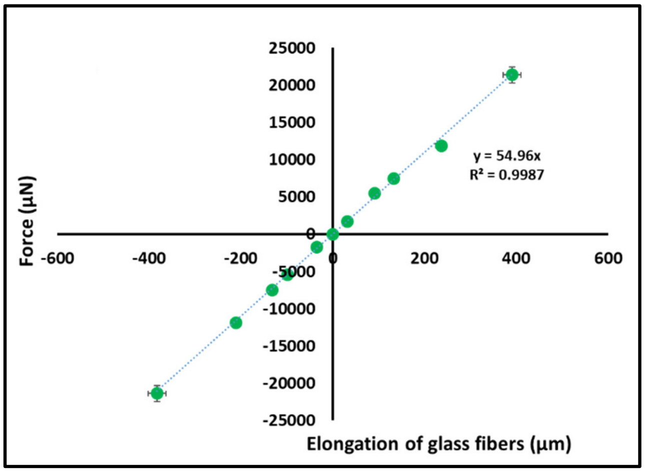

Figure S1: Determination of glass fiber (Ø 200 µm) spring constants of the force sensor. By adding additional mass to the glass fibers, we determined the glass fiber elongation and calculated the acting forces from Newton's law. Here the resulting forces are plotted as function of glass fiber elongation. The slope corresponds to the spring constant of the glass fibers which turned out to be the same for both methods.

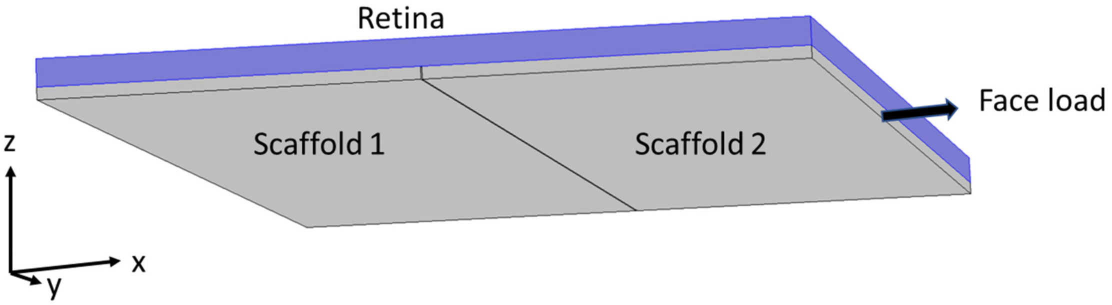

Figure S2: Sketch of the finite element simulation system: The retina (blue) was modelled on top of two adjacent titanium plates (grey) with same geometry as employed experimentally. While scaffold 1 was fixed with position constrains, a face load in x-direction was acting onto the outer surface (yz-plane) of scaffold 2 to determine the displacement of scaffold 2 and the displacement field within the retina as function of acting force and effective Young's modulus of the retina.
